# Supplementary material for: Network-Based Analysis to Identify Drivers of Metastatic Prostate Cancer Using GoNetic
Source: Cancers (Basel). 2021 Oct 21;13(21):5291. doi: 10.3390/cancers13215291 (PMC8582433; doi:10.3390/cancers13215291)
Supplement: Supplementary file 1 [file cancers-13-05291-s001.zip › cancers-1377516-Supplementary Files-for conversion.pdf]

Supplementary Files

Network-Based Analysis to Identify Drivers of Metastatic Prostate Cancer Using GoNetic

Louise de Schaetzen van Brienen, Giles Miclotte, Maarten Larmuseau, Jimmy Van den Eynden and Kathleen Marchal

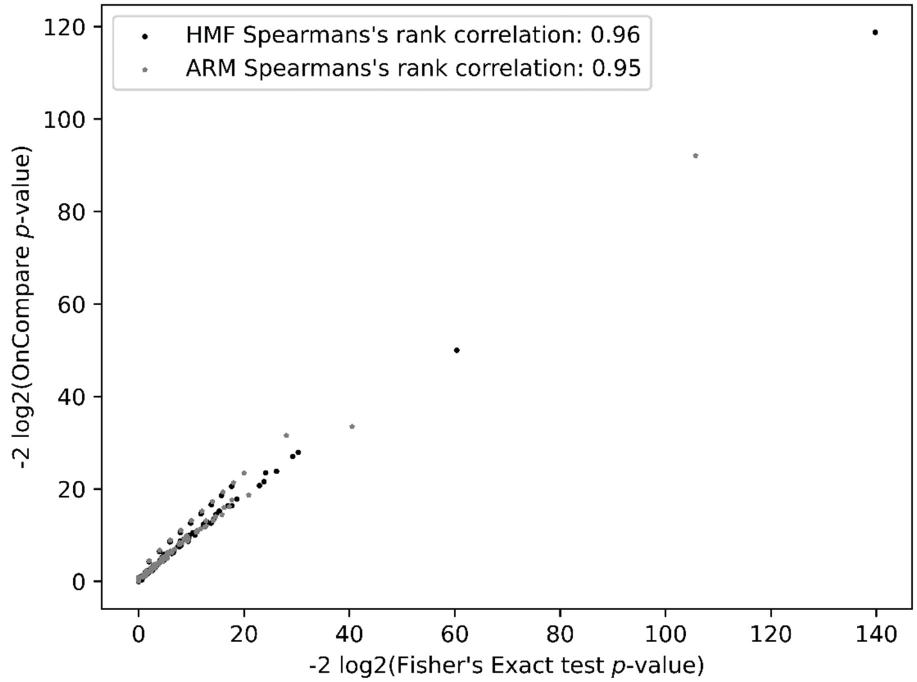

Figure S1. Correlation between the OnCompare and the Fisher's exact test *p*-values, for the comparison between the primary cohort and the HMF metastatic cohort and for the comparison between the primary cohort and the ARM metastatic cohort. No prior correction for TMB or tumor purity was performed nor were these factors taken as covariates.

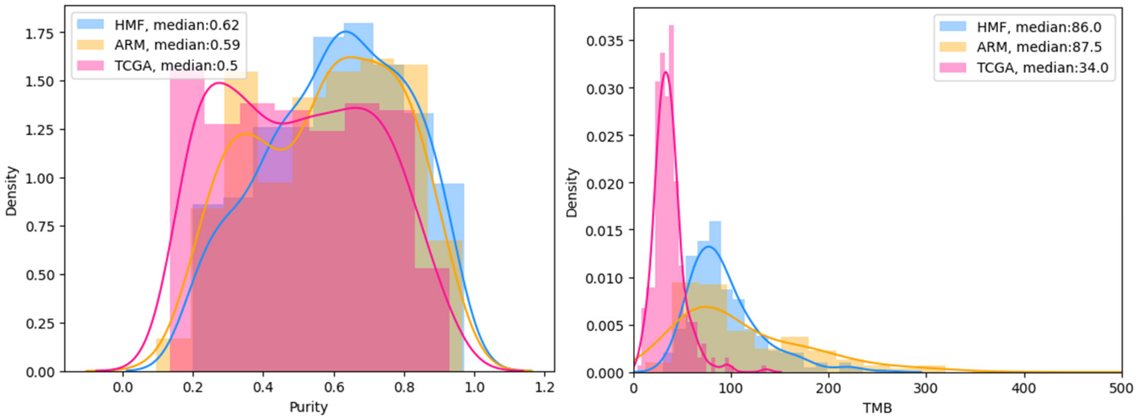

Figure S2. Comparison of the tumor purity (left) and tumor mutational burden (right) between the primary cohort (TCGA) and the two metastatic cohorts (HMF and ARM). TMB is evaluated considering all somatic variants that mapped to the regions sequenced by Armenia et al. [1] prior to applying any filtering.

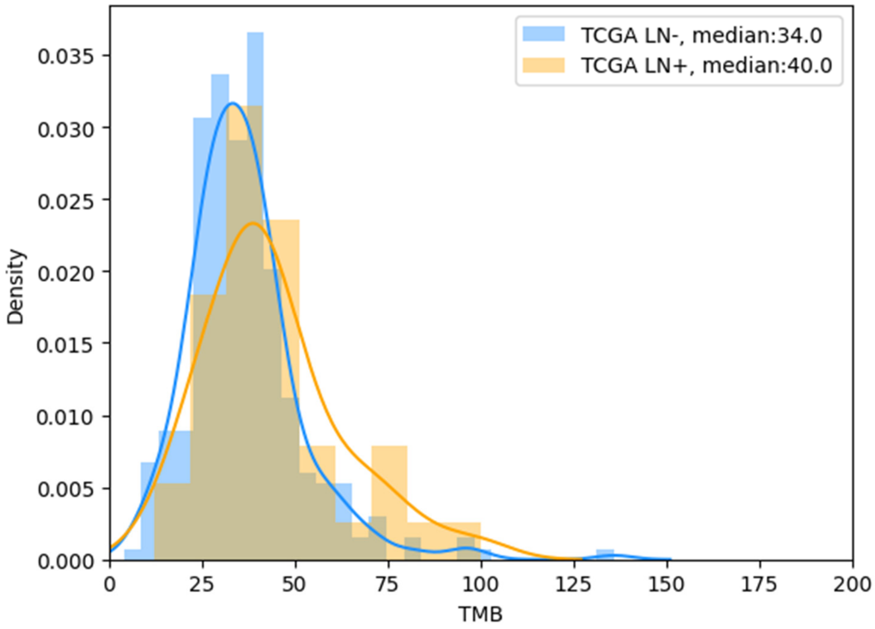

Figure S3. Comparison of the tumor mutational burden (right) between the primary cohort (TCGA) with LN- and LN+ status.

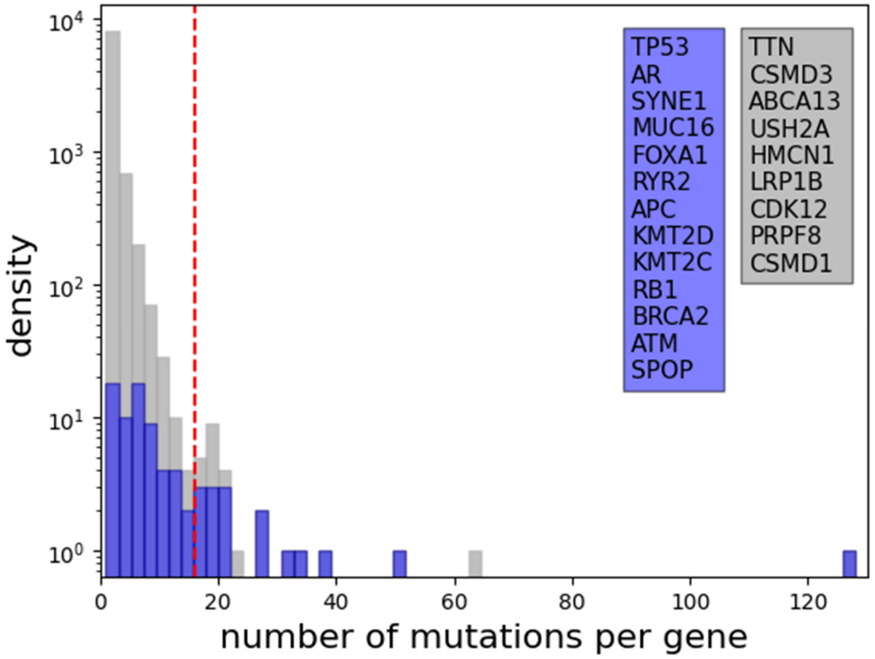

Figure S4. Histogram of the number of mutations per gene. Genes mutated in more than 5% of the samples (left to the red vertical dashed line) are shown in the boxes. In blue are genes identified by GoNetic and in gray are all the remaining genes mutated in the HMF cohort.

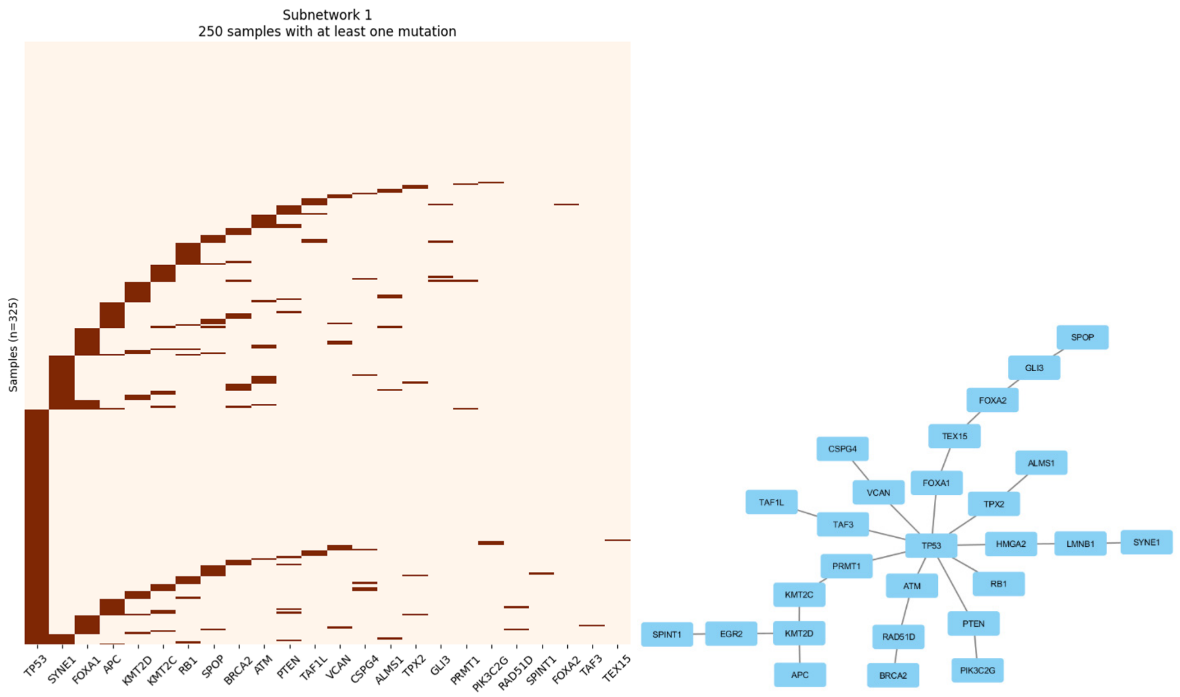

Panel A. Subnetwork 1

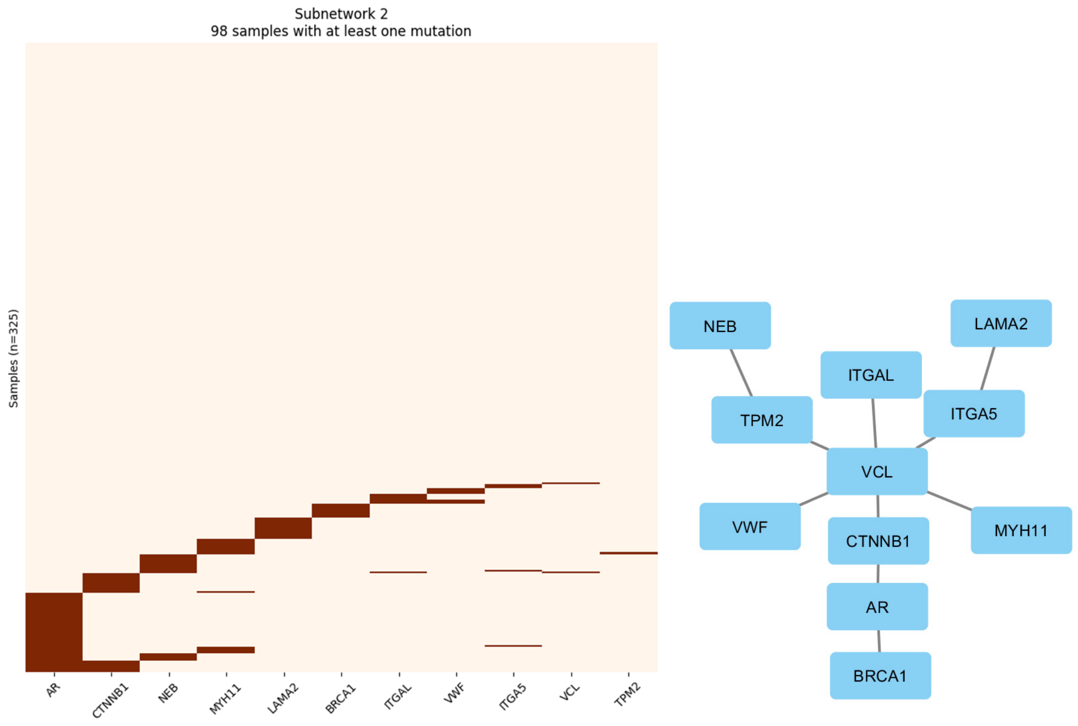

Panel B. Subnetwork 2

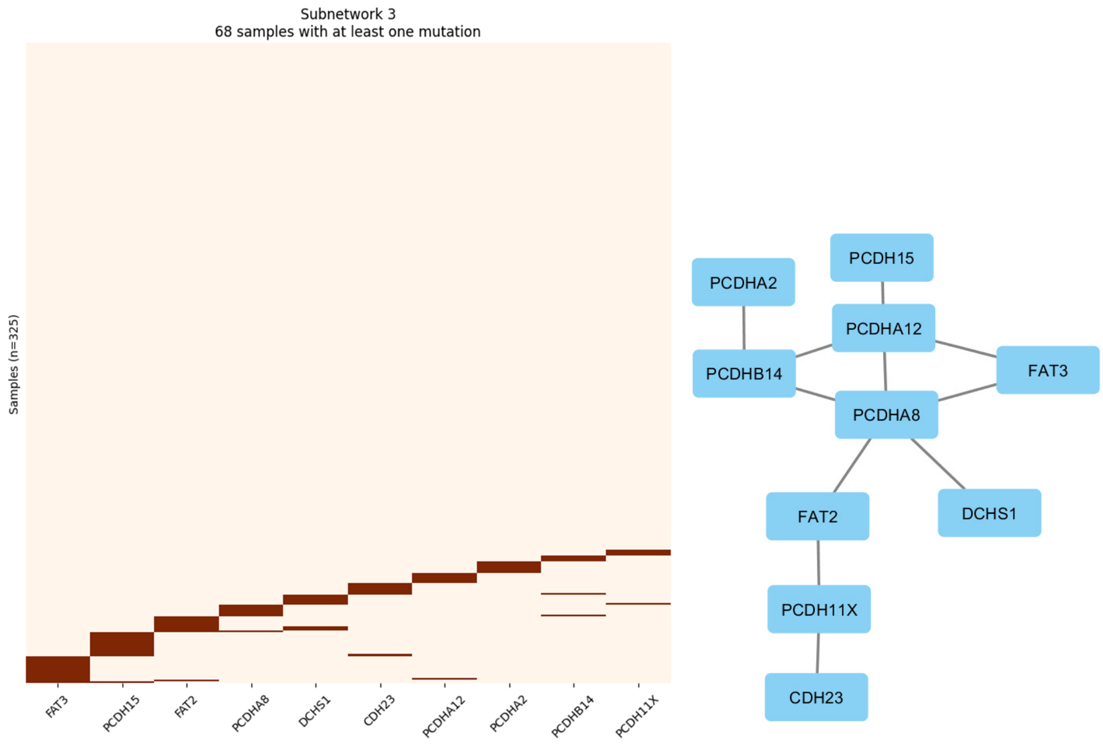

Panel C. Subnetwork 3

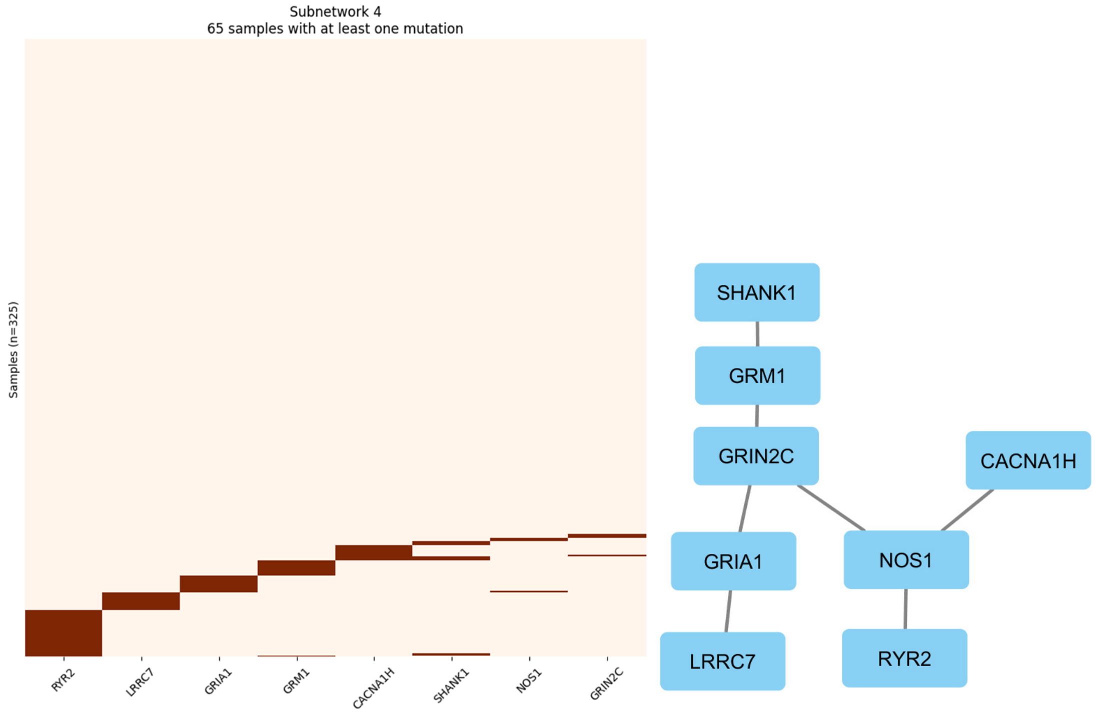

Panel D. Subnetwork 4

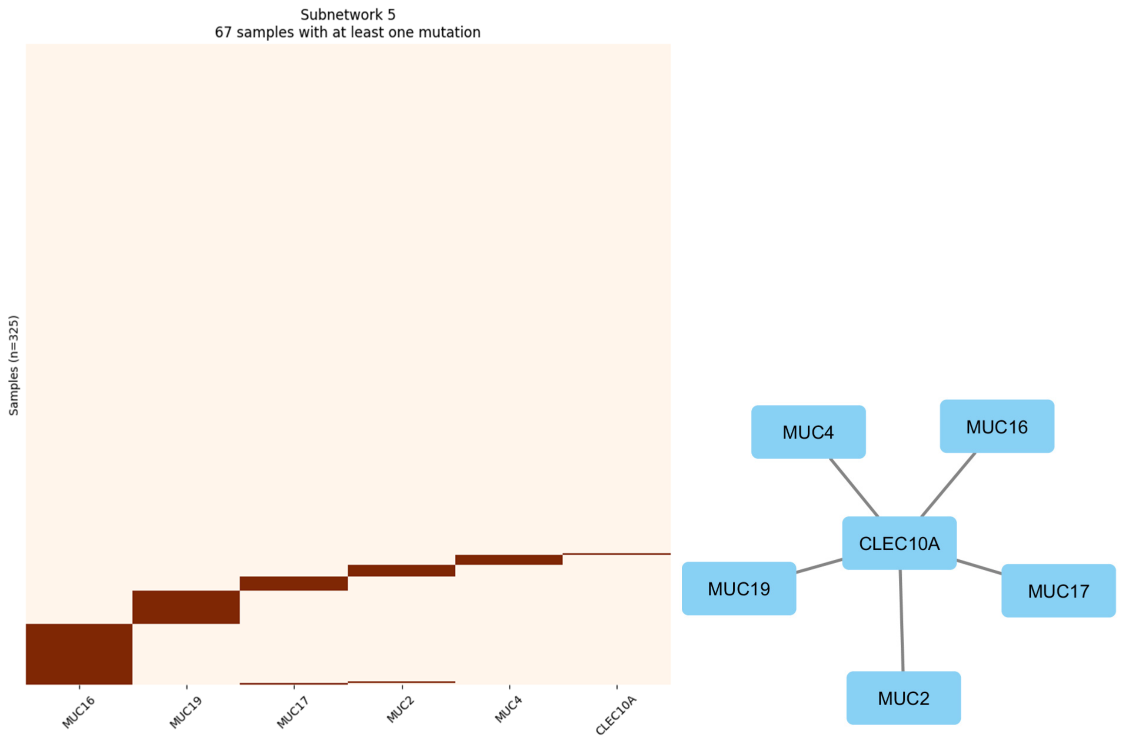

Panel E. Subnetwork 5

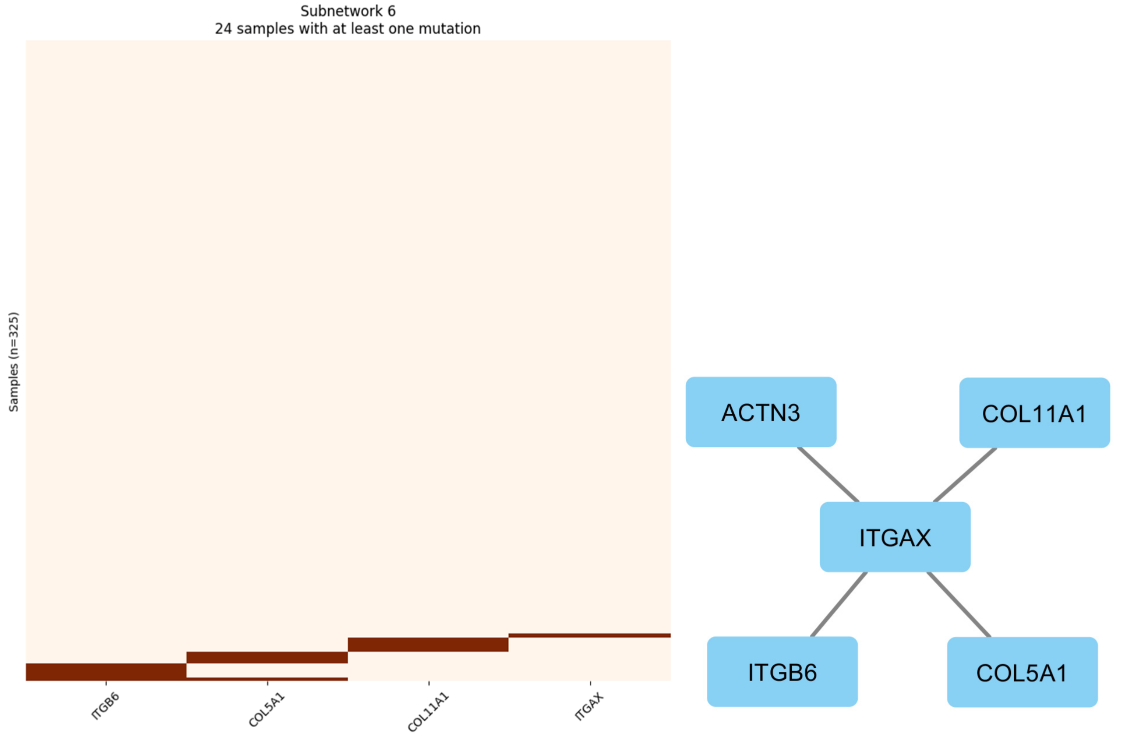

Panel F. Subnetwork 6

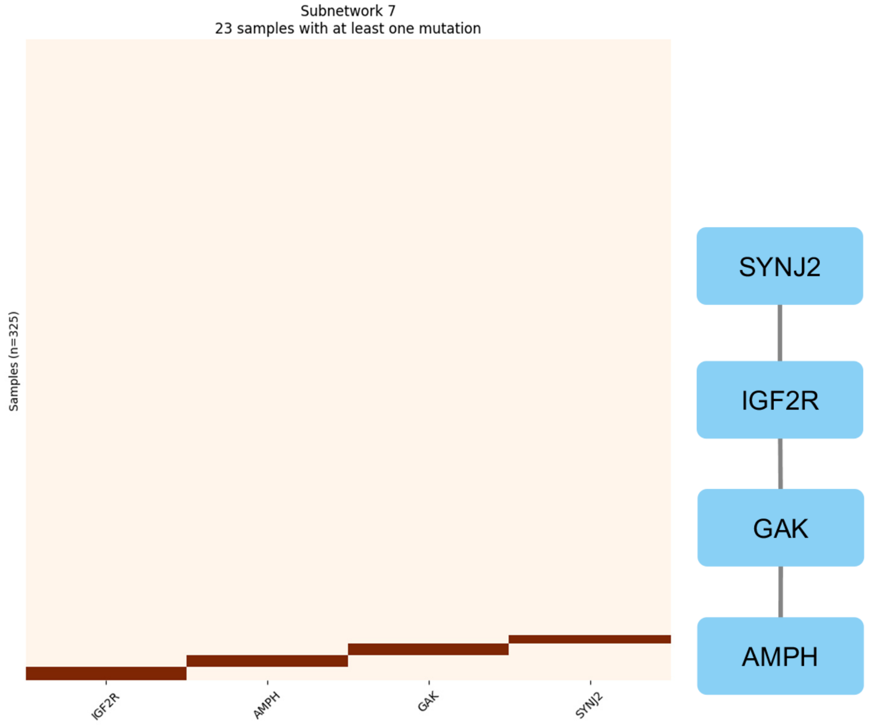

Panel G. Subnetwork 7

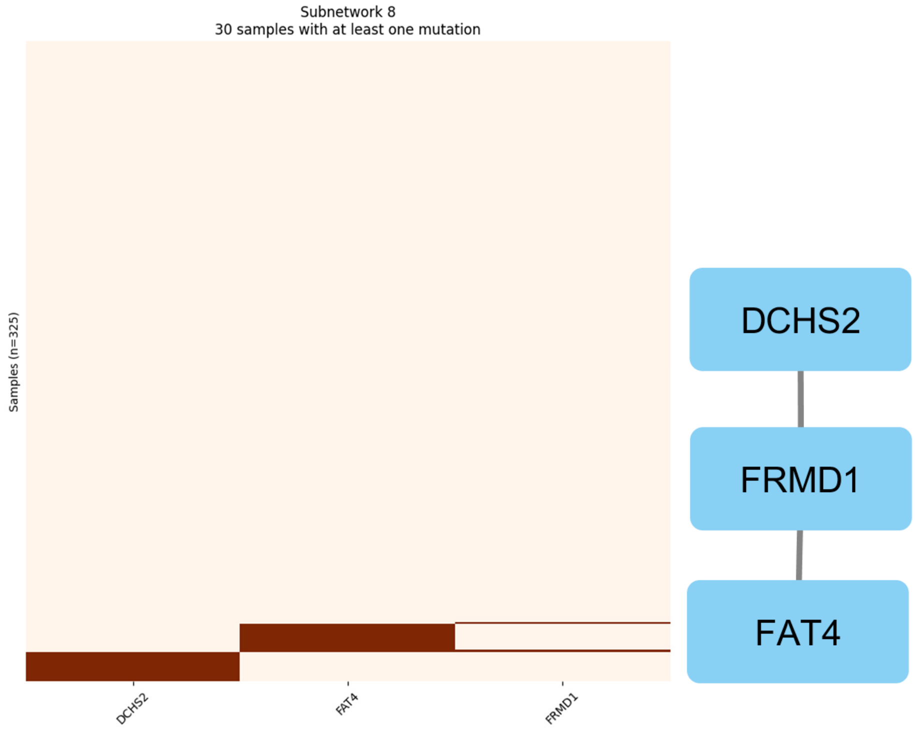

Panel H. Subnetwork 8

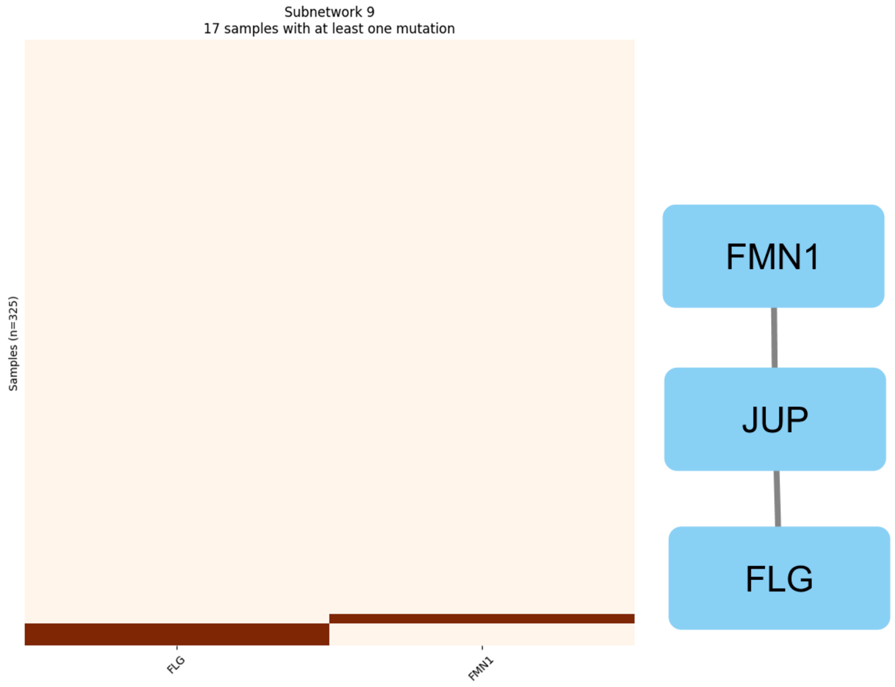

Panel I. Subnetwork 9

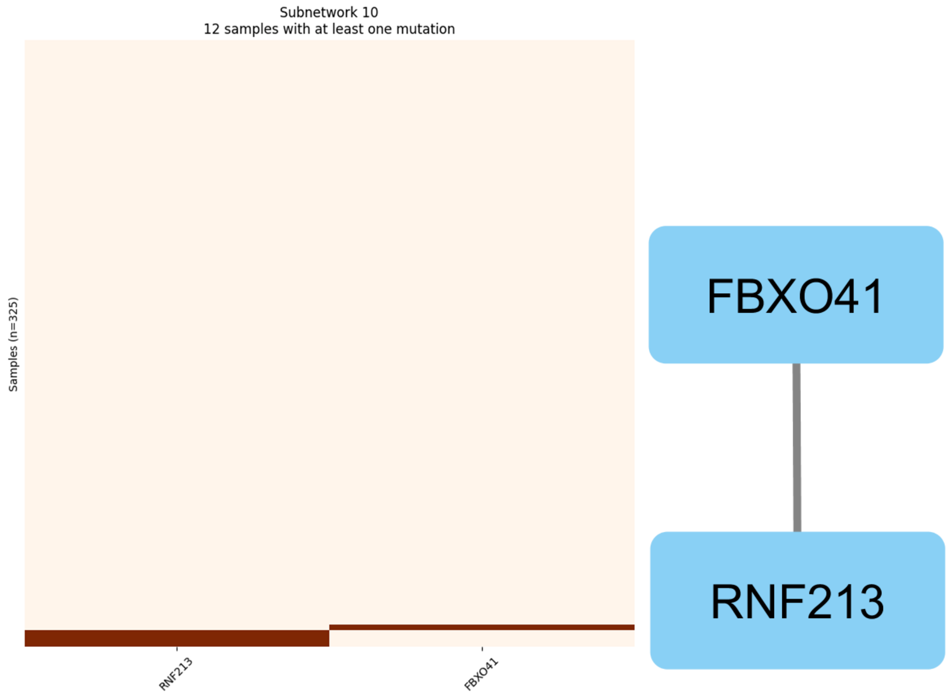

Panel J. Subnetwork 10

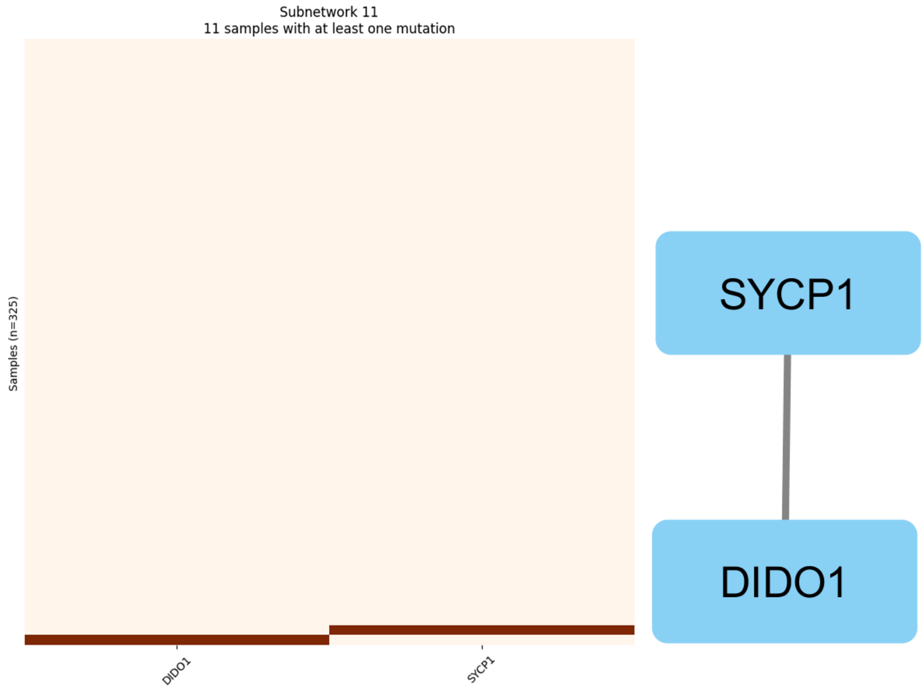

Panel K. Subnetwork 11

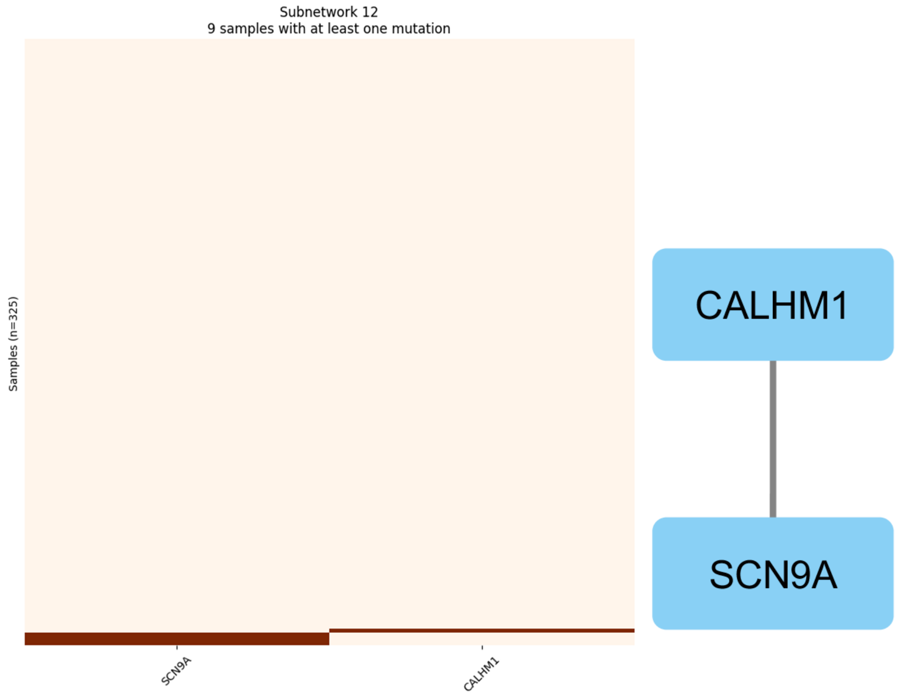

Panel L. Subnetwork 12

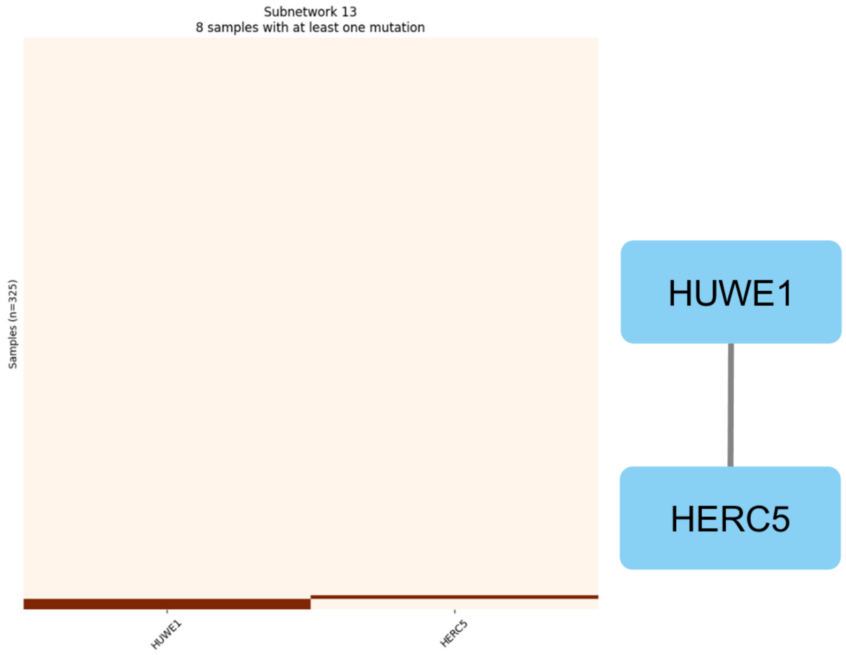

Panel M. Subnetwork 13

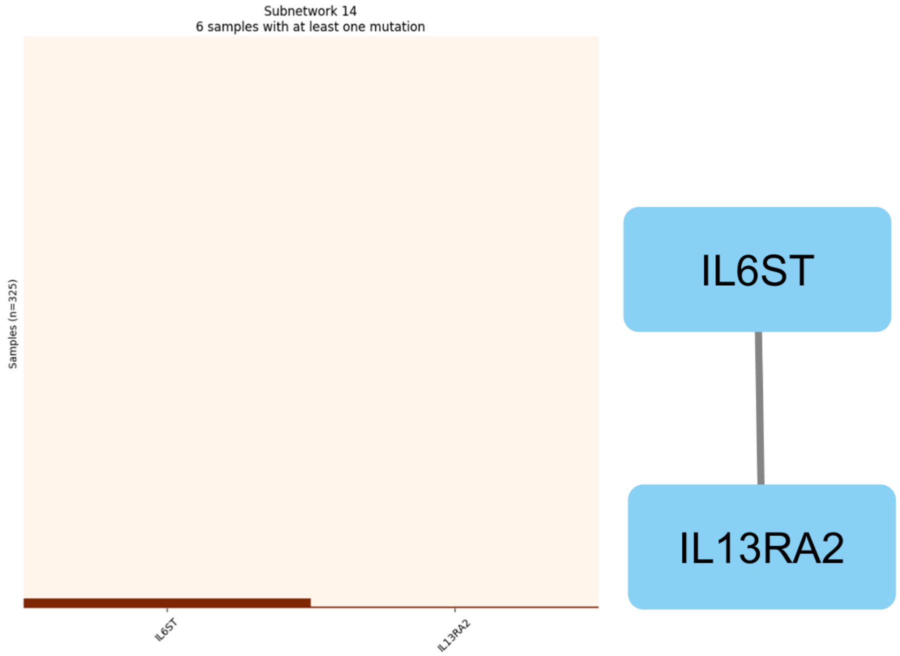

Panel N. Subnetwork 14

Figure S5. Subnetworks identified by GoNetic (left panel: Oncoprint showing the degree to which mutations in genes belonging to the same subnetwork display ME pattern, right panel: subnetwork representation).

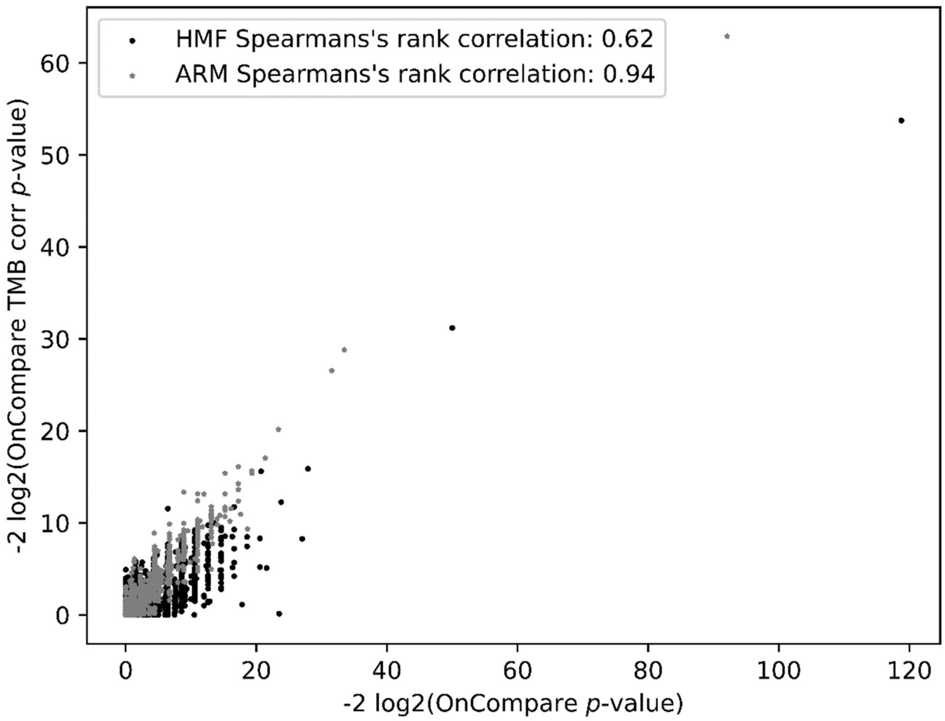

Figure S6. Correlation between OnCompare *p*-value with and without TMB correction when comparing the two metastatic cohorts to the primary cohort.

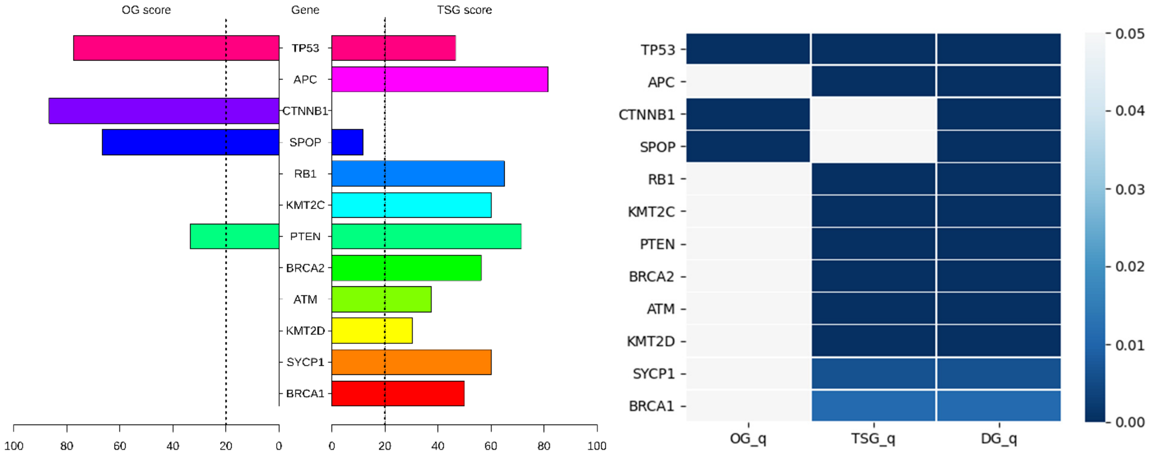

Figure S7. Results of SomInaClust analysis on the genes prioritized by GoNetic on the HMF cohort.
